# Moral Dilemmas in Contact-Based Care: The Relevance of Moral Case Deliberation for Forensic Psychiatry

**DOI:** 10.3389/fpsyt.2020.574336

**Published:** 2020-10-27

**Authors:** Sylvia Gerritsen, Guy A. M. Widdershoven, Bernard J. Bossenbroek, Yolande Voskes

**Affiliations:** ^1^Department of Ethics, Law, and Humanities, Amsterdam University Medical Center, Amsterdam, Netherlands; ^2^Fivoor, Forensische Psychiatrische Afdeling/Forensische Psychiatrische Kliniek, Rotterdam, Netherlands; ^3^GGz Breburg, Tilburg, Netherlands; ^4^Tranzo, Tilburg University, Tilburg, Netherlands

**Keywords:** forensic psychiatry, clinical ethics support (CES), moral case deliberation, safety, contact-based approach, physical intimacy, moral dilemma

## Abstract

Currently, forensic psychiatry shows a shift from a control-based to a contact-based approach. Working from contact may, however, entail new moral questions and dilemmas. How to secure safety when focusing on contact? Does contact imply being physically close to the patient, or should one refrain from intimate relations? In order to help care professionals to deal with these moral issues, clinical ethics support can be useful. A specific approach in clinical ethics support is moral case deliberation (MCD). An MCD is a structured dialogue between professionals on a moral issue they experience in practice, structured by a conversation method and guided by a facilitator. In this article, we describe the background and procedures of MCD. Furthermore, we present a case example in which care professionals reflect on the moral question of whether provision of care in forensic psychiatry may entail physical closeness. The MCD shows that an open conversation results in a better understanding of different perspectives and creates the basis for finding a joint way to proceed in the case. We conclude that MCD can enable professionals to reflect on moral issues and develop shared values in forensic psychiatry.

## Introduction

Traditionally, forensic psychiatry is known for a controlling way of working. Consequently, the use of coercion is common, often resulting in seclusion (1). However, control-based care can result in an increased level of aggression and incidents (2, 3). Interventions based on contact instead of control may contribute to less aggression and incidents (4). Moreover, a focus on contact can foster attention for patient autonomy and care. Consequently, a shift can be seen in forensic psychiatry, resulting in increased attention for reduction of coercive measures and an increasing emphasis on patient perspectives and needs (5, 6).

A contact-based approach in forensic psychiatry is promising, but the question is how to shape this in daily practice (4, 6, 7). In a complex situation, many care professionals tend to fall back on control (8). Should one refrain from control, if safety of professionals, the patient, or fellow patients is at stake? Working from contact may also involve new moral questions. Does contact entail physical proximity to the patient? If the patient is angry, should one try to calm him by holding him? If a patient is sad, should he be comforted? How far does a contact-based approach in forensic psychiatry go? These questions can cause moral tensions and doubts among care professionals.

How can professionals in forensic psychiatry be assisted in dealing with moral tensions involved in working from contact? One way to do this is to provide clinical ethics support (CES), fostering reflection on difficult moral issues and providing professionals with tools to handle them. In mental health care organizations, the use of CES is common. However, compared to general psychiatry, CES is not well-established in forensic psychiatry (9). A specific approach in CES is moral case deliberation (MCD). In an MCD meeting, care professionals jointly reflect on a moral dilemma experienced by one of the participants, guided by a facilitator who uses a structured conversation method (10, 11). In this article, we describe the background and procedures of MCD. We also present an example in which professionals in forensic psychiatry reflected on a case in practice. Finally, we discuss the relevance of MCD for dealing with moral tensions in forensic psychiatry.

## Materials and Methods

MCD is a specific approach in CES that aims to foster systematic reflection on moral questions (10). MCD has a theoretical background in pragmatic hermeneutics and dialogical ethics (12). This background manifests itself by an emphasis on a concrete, practice-oriented case, which is experienced by the participating care professionals themselves, or is easy to envision for them. In an MCD, care professionals engage in a dialogue, aiming at openness to and exchange between perspectives. This can result in a deeper understanding of the concrete moral issue. Overall, the use of MCD aims for a joint learning process of care professionals in which awareness and mutual understanding are fostered (10). Over the years, increased attention has been paid to the use of MCD in Dutch care settings, especially in psychiatry (13).

A widely used conversation method used for MCD is the dilemma method (11). In this method, care professionals are stimulated to reflect on their own moral experiences in practice. Jointly, care professionals with a multidisciplinary background reflect on a case that is brought in by one of the participants. Participants proceed through a series of steps under the guidance of an independent, trained facilitator. In the dilemma method, the situation is defined in terms of two options, two possible actions that are mutually exclusive, and both have moral disadvantages. By doing so, the moral problem becomes concrete for the participants. As a result, care professionals can place themselves in the situation and make their own moral considerations explicit (11). The purpose of this method is to reflect on each other's perspectives in order to come to a new and richer view of the situation. An overview of the steps of the dilemma method is as follows:

### Steps of the Dilemma Method (14)

1. Presentation of the case
2. Formulating the moral dilemma
3. Questions for clarification
4. Analysis of the perspectives in the case
5. Exploring alternatives
6. Making an individual judgment
7. Dialogue
8. Evaluation

## Results

### A Case Example

In this section, we describe an MCD on a ward of a medium-security level forensic care organization in the Netherlands. The MCD was organized because the team was confronted with a complex situation in which it was difficult to prevent escalation. A patient at the ward, S., was agitated, and two care professionals involved in taking care were unable to calm the patient. They did not know how to establish contact with the patient and provide adequate care. By means of a joint reflection, the team aimed to gain more insight into the situation and to find ways to deal with this and comparable situations. In total, 11 care professionals participated with different professions: seven forensic mental health nurses (with a background as nurse or social therapist) and a social worker, psychologist, psychiatrist, and team manager. The duration of the MCD was 120 min. Below is an elaboration of the MCD, following the steps of the dilemma method. Because of privacy reasons, the case has been modified, and all identifying details have been removed, including the gender of the patient.

#### Presentation of the Case

One of the two forensic mental health nurses involved in the dilemma presented the case. S. is an adult diagnosed with autism and has a low IQ, resulting in S. having a developmental age of a child. S. was referred from another forensic psychiatric hospital in the Netherlands, where S. caused a serious incident, involving verbal aggression and serious physical threats to others and to oneself. Consequently, S. was recently admitted to the present forensic psychiatric hospital.

Because the staff wanted to reduce the stimuli, S. had to remain in a private room most of the day. Four times a day, S. was allowed to go to the living room for half an hour. During these moments, S. was supervised by two care professionals to prevent escalations with fellow patients. This was considered challenging. S. was now 3 weeks on the ward, was often angry, and refused to be supervised by certain care professionals. As the needs of S. differed from the usual population that remains at this ward, care professionals tried to adjust the provision of care.

One morning, two forensic mental health nurses went to the room of S. to wake S. After an hour, they returned to guide S. to the living room. The care professionals noticed that S. acted in a peculiar way, and they asked what was wrong. S. did not respond, became angry, and left the room to go to the laundry room to wash some clothes. S. did not manage to get the washing machine running and became frustrated. S. went to the smoking room, the computer room, the kitchen, and so on. Despite various attempts of the care professionals, it appeared impossible to get in touch with S. S. fell to the ground and started to rage wildly with both legs. As S. was lying on the floor, the two care professionals were in doubt what to do. Should they force S. to go back to the private room, in order to calm down, or should one of them sit down next to S. and try to comfort S.?

#### Formulating the Moral Dilemma

Based on the explanation of the situation and the doubts of the two professionals, the dilemma was formulated as follows:

A: I force S. back to the private room
B: I sit down next to S. and physically comfort S.

After formulating the dilemma, the participants were asked to make explicit negative consequences of both options. Option A would probably lead to resistance. S. would not cooperate and refuse to go to the private room. As it was likely that S. might get more angry, it would take at least six care professionals to take S. to the room. Consequently, the relation between S. and the care professionals would be damaged. While S. would have to be locked up in the room, contact would be impossible. Also, S. would be confined to the room and deprived of freedom.

Option B would entail that one of the care professionals would sit down next to S. This would make the care professional vulnerable and potentially at risk, as S. was angry and moving wildly. Touching S. might work counterproductive and result in an increase of tension and possible physical risks. Another negative consequence might be that touching S. could result in an uncomfortable feeling, both in the professional who would do so in order to comfort and in other professionals. Finally, care professionals mentioned that other patients might find it unfair as they would receive less attention.

Professionals were also asked to define the moral question central in this dilemma. They formulated the following question: “How (physically) close are you allowed to be in the provision of forensic psychiatric care?”

#### Questions for Clarification

Next, participants were asked to place themselves in the position of the two care professionals. In order to do so, they might need more information. Thus, all participants were invited to ask questions about the situation. This resulted in a further explanation: the situation took place in the kitchen; other patients watched the situation, and apart from the two care professionals, there were two trainees and a facility worker present at the ward.

#### Analysis of the Perspectives in the Case

In the next step, participants were invited to consider what was important for the people involved in the case. They focused on the perspective of the two care professionals, S., and other patients at the ward. In order to specify what was important for each perspective, they were asked to formulate values (moral motivations) and for each value the associated norm (rule for action). In this section, we will elaborate on the most important values and norms that were mentioned. For a schematic overview of all the values and norms per stakeholder, see Table 1.

**Table 1:** Schematic overview of all the values and norms per stakeholder.

**Perspective**	**Value**	**Norm**
Care professionals	Safety	“I should work de-escalating” (care professional A, sociotherapist)
		“I have to provide safety for the patient” (care professional B, nurse)
		“I should avoid danger” (care professional C, nurse)
		“I have to protect my own boundaries” (care professional D, nurse)
	Rest	“I have to limit the amount of stimuli” (care professional E, psychiatrist)
	Good care	“I have to make contact” (care professional F, nurse)
	Professionalism	“I shouldn't make physical contact with patients” (care professional G, sociotherapist)
	Predictability	“I want to be on the same page with my colleagues and with the patient” (care professional H, social worker)
S. (patient)	Clarity	“I should understand”
	Equality	“I would like the same approach from everyone, structure”
	Safety	“I need to know what is about to happen”
	Trust	“I have to be able to trust the staff, that they do what we agreed upon”
	Autonomy	“I have to be able to express myself (unleash emotions)”
	Empowerment	“I should be able to be in the living room, to do my laundry whenever I want”
Other patients at the ward	Equity	“We should receive the same treatment”
	Attention	“We want attention”
	Safety	“We don't want to risk the patient attacking us”
	Rest	“We don't want tension on the group”
		“I already have enough on my mind”

First, the care professionals analyzed the perspective of the two care professionals involved in the case. They all regarded safety as an important value. However, while placing themselves in the position of the nurses in the case, the participants translated the value of safety into different norms. For one participant, realizing safety implied: “I should work de-escalating” (care professional A, sociotherapist); another participant translated the value of safety into “I should avoid danger” (care professional B, nurse); a third participant proposed as a norm: “I have to provide safety for the patient” (care professional C, nurse). Next, the value of care was identified as relevant. Care professional F, a nurse, translated this value into the norm: “I have to make contact.” Also, professionalism was regarded as important; one of the participants formulated as corresponding norm: “I shouldn't make physical contact with patients” (care professional G, sociotherapist).

Second, participants placed themselves in the perspective of S. In contrast to the analysis of the perspective of the care professionals, which resulted in differences between participants, they agreed on relevant values and norms for the patient. They regarded clarity to be an important value for S., which was translated into the norm “I should understand.” Also, safety was seen as important, which gave rise to the norm “I need to know what is about to happen.” Participants also mentioned the values autonomy (“I have to be able to express myself and unleash emotions”) and empowerment (“I should be able to be in the living room, to do my laundry whenever I want”).

Third, participants identified important values for other patients staying at the ward. Again, they agreed on relevant values and norms. They specifically mentioned equity (“we all should receive the same treatment”) and safety (“we don't want to risk the patient attacking us”).

#### Exploring Alternatives

After the participants had identified the values and norms relevant for the different people involved, they were asked to mention possible alternatives. In this step, participants were stimulated to think creatively and let go of standard solutions. Various alternatives were identified, for instance, “ignore S.,” “sing/turn on children songs,” and “put S. in the seclusion room.”

#### Making an Individual Judgment

The next step was to make an individual moral judgment. Each participant was asked to consider for themselves whether it was morally right to force S. to the private room (A) or sit down next to S. and physically comfort S. (B). Furthermore, care professionals were asked to indicate which value was most important to them in this decision, what they envisioned as the consequential damage of their decision, and how they would try to diminish or repair the damage.

Some care professionals considered it morally correct to do A, which was to force S. to the private room based on the value of safety. A negative consequence of this option was that they would put their own safety first. Also, they would not help S. and damage the values of trust and freedom. To diminish these damages, they would try to clearly communicate and explain the decision to S. This would imply that they would need other colleagues to bring S. to the private room.

Other care professionals considered it morally correct to do B, which implied to sit down next to S. and physically comfort S. based on the value of good care. According to one of the care professionals, S. was actually a child in an adult body. The care professionals choosing this option also saw disadvantages. For example, some care professionals might feel uncomfortable to physically touch a patient. It would also deviate from the usual care at the ward. An extra challenge was that S. would accept certain actions from one professional but not from another. And S. might respond negatively, which would result in danger. To diminish this risk, some care professionals suggested to talk to S. and to explain what they were about to do. To be able to realize this option, they mentioned that they would need sufficient care professionals nearby whom they trust.

#### Dialogue

After exchanging individual judgments, participants investigated similarities and differences. Similarities entailed the importance to provide good care, to get in contact with S., and to foster safety. There were, however, differences in how to realize these values, especially regarding safety. There were also different views on whether or not to come physically close to and touch the patient.

In a dialogue, the focus is not on defending one's own position, but on trying to understand the position of the other and its relevance for oneself. How can the action proposed by someone else be helpful in realizing one's own values? Can different ways to realize the value of safety be relevant, given the situation? The professionals who went for option B explained that the anger of S. was caused by feelings of insecurity and that forcing S. would probably make this feeling even more pronounced. On the other hand, comforting S. might reduce the fear for not being in control. This might result in de-escalation and more safety for everyone. Of course, the risk remained that this would not work; thus, they proposed to closely monitor the situation and go for option A if necessary. Having option A as a last resource supported the views of those who are afraid that other means might not work and therefore chose this option in their individual judgment.

Next, the question whether physical comfort can be part of forensic psychiatric care was examined in dialogue. Everyone agreed that a professional attitude requires some distance. Yet, in care for children, physical contact is important. Because S. reacted as a child, comforting S. seemed to be in order. Morally speaking, not every care professional could be expected to take this role. Thus, those who did not regard touching a patient as part of their professional identity should not feel obliged to do so. It was agreed that those professionals who tended to respond to S. by sitting down and touching might try to do so, in order to see whether this would work. Thus, the decision was made to first try option B, with professionals positive about touching sitting next to S.; if this would not work, option A would serve an alternative solution, and S. would be brought to the private room.

A subsequent topic for investigation was how to secure good cooperation, as not all professionals responded in the same way. The participants wanted to prevent that the team would become polarized, with a distinction between those who are willing to provide physical comfort and those who do not. The conclusion was that one should be open about this, both to each other and to the patient. As patients' needs are different, and professionals' attitudes diverge, it would be important to work together in providing the best care for the individual patient. This should not mean that everyone would provide the same care, but that all would agree on the division of care tasks and support each other, whatever care they individually would provide.

#### Evaluation

The participants evaluated the deliberation positively. They noticed that the moral concerns and motivations of care professionals for certain decisions, including underlying values and norms for action, had become clear. The participants decided to organize a follow-up meeting. In this meeting, a joint crisis plan would be made. They agreed that everyone should be informed about the plan and feel comfortable with it. The personal limits of care professionals in regard to physically touching this patient should be respected in this plan.

## Discussion

The shift in forensic psychiatry from control to contact leads to moral concerns and questions in daily practice. MCD can support professionals in dealing with moral issues, by fostering joint reflection and dialogue. By making explicit core values as well as various views on how to realize them, MCD can create a basis for more mutual understanding and better cooperation. The case example shows that having a dialogue on how to foster safety and whether to provide physical comfort results in finding new ways to deal with a complex situation, doing justice to the concerns, and experiences of all parties involved.

The case example shows that solutions should be fitted to the specific situation. S. required another approach than other patients. This approach could not be provided by all professionals. The conclusion that professionals should take into account the specific situation is in line with pragmatic hermeneutics, underlining the importance of focusing on concrete, practical problems (10). It is important that care professionals reflect on what is best for a specific patient and on their own boundaries and do not blindly follow a framework that tells them what they should or should not do (15).

This study reveals that in difficult situations, care professionals can be tempted to take control and diminish possible risks (8). Therefore, it is important that care professionals are open to various options and learn how to achieve alternative values. This is also concluded by Steinert: “Eventually, it is necessary to further develop the current practices, away from safety measures imposing severe distress to patients and staff toward interventions, which integrate relationship-building, trust, and the search for agreement into every coercive approach” (16). However, working from contact and dealing with the tensions involved require more than just practical tools. It calls for a change in culture and attitude of care professionals (17). MCD can help to shape this new culture by supporting reflection on values and developing shared way of dealing with difficult situations.

The use of MCD was shown to be useful for the care professionals in the case example. Care professionals concluded that they gained more awareness of each other's motives and more insight into their own boundaries as a professional. They also appreciated the structure, which in their perception created more mutual understanding and a direction for next steps to take. One of the conclusions in this MCD was that everyone should be respected in their views concerning whether or not to physically comfort a patient and to discuss this openly. These findings are in line with the conclusions of Weidema et al.: “Moral case deliberation is related to mutual support and consultation; improves communication, quality of care, and connection; stimulates critical reflection and brings assertiveness or emancipation to the nursing profession” (18). Other studies likewise emphasize the importance of sharing experiences through reflection and having a dialogue on moral issues in forensic practice (8, 9, 14, 19, 20).

In this study, we focused on a single MCD to give a concrete and in-depth description of the method and discussed dilemma. In line with case study research, the results are not generalizable; yet, they are transferable to other contexts as they can provide suggestions for interpretation of experiences and for practice improvement (21). This study is in line with other studies, signaling difficulties in comparable transitions within forensic psychiatry, and a need for support and supervision to deal with this (22, 23). Dilemmas of whether or not to touch a patient can arise in this transition, as is also noticed by Weiskopf (24). We recommend follow-up research on dilemmas experienced in the transition from control-based to contact-based care in forensic psychiatry and will undertake such studies in the Netherlands.

CES, in general, and MCD, in particular, may help professionals in dealing with dilemmas in forensic psychiatric care. The case example presented here shows that an open conversation results in a better understanding of different perspectives and creates the basis for finding a joint way to proceed in the case. We conclude that MCD can enable professionals to reflect on moral issues and develop shared values in forensic psychiatry.

## Data Availability Statement

The original contributions presented in the study are included in the article/supplementary material, further inquiries can be directed to the corresponding author/s.

## Ethics Statement

Ethical review and approval was not required for the study on human participants in accordance with the local legislation and institutional requirements. Written informed consent for participation was not required for this study in accordance with the national legislation and the institutional requirements.

## Author Contributions

SG was the facilitator of the MCD. BB was team manager of the ward where the MCD took place. All authors participated in the analysis of the MCD, discussions about the analysis and results, revising the work critically and read, revised, and approved the final manuscript.

## Conflict of Interest

The authors declare that the research was conducted in the absence of any commercial or financial relationships that could be construed as a potential conflict of interest.
